# RETRACTION: Effective Innovative Technologies and One Health Strategies in Mitigating Aflatoxin Contamination in Peanut Oil: A Systematic Review and Meta‐Analysis

**DOI:** 10.1002/fsn3.71758

**Published:** 2026-04-15

## Abstract

**RETRACTION**: YenewC. , MekonenS. , AmbeluA. , YeshiwasA. G. , "Effective Innovative Technologies and One Health Strategies in Mitigating Aflatoxin Contamination in Peanut Oil: A Systematic Review and Meta‐Analysis," Food Science and Nutrition 13, no.2 (2025): e70062. 10.1002/fsn3.70062.39974508 PMC11837028

The above article, published online on 19 February 2025 on Wiley Online Library (wileyonlinelibrary.com), has been retracted by agreement with the Editor‐in‐Chief Y. Martin Lo, the authors and Wiley Periodicals LLC. A third party reported that a significant number of references in this article were either incorrect or nonexistent. All parties have determined that the large number of incorrect references constitutes a major error and leaves significant portions of the article unsubstantiated. This retraction has been agreed to because these errors fundamentally compromise the content and conclusions of the article.

**RETRACTION**: C. Yenew , S. Mekonen , A. Ambelu , A. G. Yeshiwas , "Effective Innovative Technologies and One Health Strategies in Mitigating Aflatoxin Contamination in Peanut Oil: A Systematic Review and Meta‐Analysis," Food Science and Nutrition 13, no.2 (2025): e70062. 10.1002/fsn3.70062.39974508 PMC11837028

The above article, published online on 19 February 2025 on Wiley Online Library (wileyonlinelibrary.com), has been retracted by agreement with the Editor‐in‐Chief Y. Martin Lo, the authors and Wiley Periodicals LLC. A third party reported that a significant number of references in this article were either incorrect or nonexistent. All parties have determined that the large number of incorrect references constitutes a major error and leaves significant portions of the article unsubstantiated. This retraction has been agreed to because these errors fundamentally compromise the content and conclusions of the article.

